# miR-144-3p, a tumor suppressive microRNA targeting ETS-1 in laryngeal squamous cell carcinoma

**DOI:** 10.18632/oncotarget.7025

**Published:** 2016-01-27

**Authors:** Si-Yi Zhang, Zhong-Ming Lu, Ye-Feng Lin, Liang-Si Chen, Xiao-Ning Luo, Xin-Han Song, Shao-Hua Chen, Yi-Long Wu

**Affiliations:** ^1^ Department of Otorhinolaryngology, Guangdong General Hospital and Guangdong Academy of Medical Sciences, Guangzhou, Guangdong Province, China; ^2^ Guangdong Lung Cancer Institute, Guangdong General Hospital and Guangdong Academy of Medical Sciences, Guangzhou, Guangdong Province, China

**Keywords:** biomarker, laryngeal squamous cell carcinoma, migration, miR-144-3p, prognosis

## Abstract

Regional lymph node metastasis and distant metastasis are critical in the prognosis of laryngeal squamous cell carcinoma (LSCC). This study investigated the roles of miR-144-3p and E26 transformation specific-1 (ETS-1) in the invasion and migration of LSCC cells. The effects of miR-144-3p and ETS-1 on FaDu and Hep2 cell growth, migration and invasion were determined. Suppression of ETS-1 by miR-144-3p was confirmed using luciferase assays; the effects of ETS-1 silencing were determined using a xenograft tumor model. The expression of ETS-1 was analyzed in 71 paraffin-embedded tissue biopsies and eight fresh frozen biopsies obtained from LSCC patients. miR-144-3p inhibited the growth, invasion and migration of FaDu and Hep2 cells in part through suppression of epithelial-mesenchymal transition as determined by increased E-cadherin and α-catenin and reduced fibronectin and vimentin expression. Additionally, ETS-1 is a molecular target of miR-144-3p, and silencing ETS-1 expression inhibited FaDu and Hep2 cell invasion and migration as well as reduced Hep2 xenograft tumor volume. In LSCC, the expression of ETS-1 is upregulated with disease progression, and higher ETS-1 expression, which was negatively associated with miR-144-3p levels, adversely corresponded with prognoses. Thus, upregulated ETS-1 levels may promote LSCC metastasis, resulting in poor patient prognosis.

## 1 INTRODUCTION

Head and neck squamous cell carcinoma (HNSCC) is the sixth most abundant cancer worldwide [1]. Laryngeal squamous cell carcinoma (LSCC) accounts for a quarter of all HNSCCs, and its incidence continues to rise [2–3]. Although great progress has been made in the treatment modalities for LSCC, the five-year survival rate of patients has not significantly improved [4]. Regional lymph node metastasis and distant metastasis are important factors that negatively impact the survival of LSCC patients [5].

microRNAs (miRNAs) play important roles in the occurrence and development of human cancers, including HNSCCs [6–9]. In a previous study, we found that the expression of miR-144-3p was downregulated in LSCC [10], which is similar to previous studies [11, 12]. However, in nasopharyngeal carcinoma, miR-144 is an onco-miRNA that is upregulated [13]. At present, the role of miR-144 in LSCC had not been elucidated.

We previously predicted that the transcriptional oncoprotein, E26 transformation specific-1 (ETS-1), is a putative molecular target for miR-144-3p using PicTar, miRanda, and TargetScan [10]. In both acute lymphoblastic leukemia cells and acute myeloid leukemia cells, an ETS-1/Smad4 complex induces antifolate resistance [14]. Moreover, accumulation of ETS-1 in breast cancer cells induced anchorage-independent growth [15]. A role for ETS-1 in targeted drug delivery against metastatic cells has also been suggested [16]. Furthermore, Calli et al. [17] reported that ETS-1 expression is upregulated in LSCC and may play a role in invasion. Thus, this study was undertaken to explore the effects of miR-144 on the invasion and metastasis of LSCC. These studies may form the basis for further analysis of the predictive potential of analyzing miR-144-3p in LSCC.

## 2 RESULTS

### 2.1 miR-144-3p inhibits cellular migration and invasion

**Figure 1:**
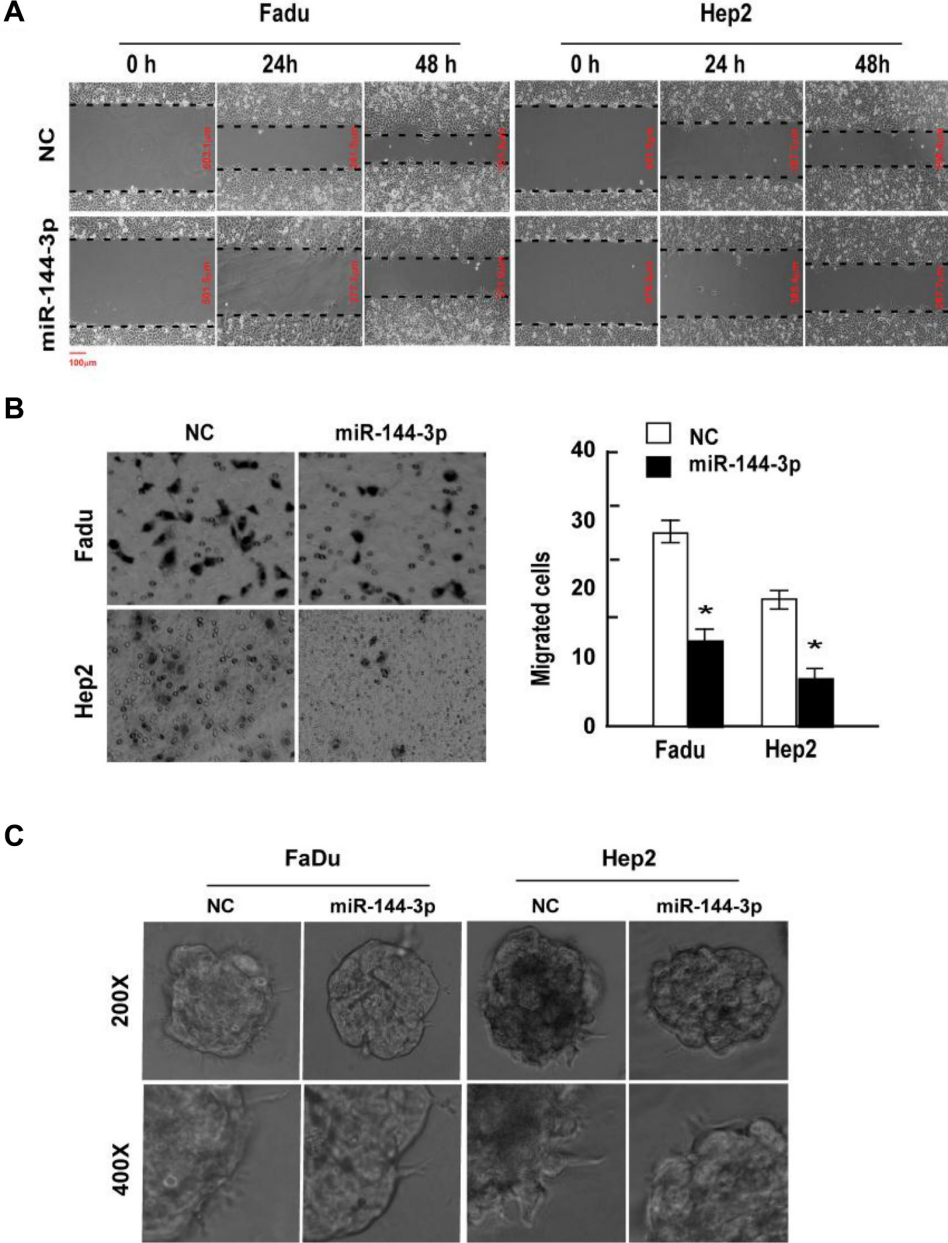
miR-144-3p inhibits FaDu and Hep2 cell invasion and migration FaDu and Hep2 cells were transfected with miR-144-3p mimics, and cell invasion and migration was determined using a (**A**) wound-healing assay, (**B**) Transwell^®^ invasion assay, and (**C**) 3D-culture test. Values were presented as mean and standard deviation (SD). (B, right panel) *p < 0.05 compared with the NC group.

FaDu and Hep2 cells were transfected with miR-144-3p mimics, which significantly increased miR-144-3p levels (Supplementary Figure 1). Following transfection with miR-144-3p mimics, cell invasion and migration were evaluated in a wound-healing assay, Transwell^®^ invasion assay, and 3D-culture test (Figure 1). miR-144-3p mimics inhibited FaDu and Hep2 cell migration as compared to the negative control (NC) cells (Figure 1A). In the invasion assay, miR-144-3p mimics significantly suppressed cell invasion (*p* ≤ 0.002; Figure 1B). Analysis of 3D cultures revealed that upregulation of miR-144-3p resulted in fewer and shorter processes than observed in the NC cells (Figure 1C), indicating a reduced propensity for invasion and migration.

**Figure 2:**
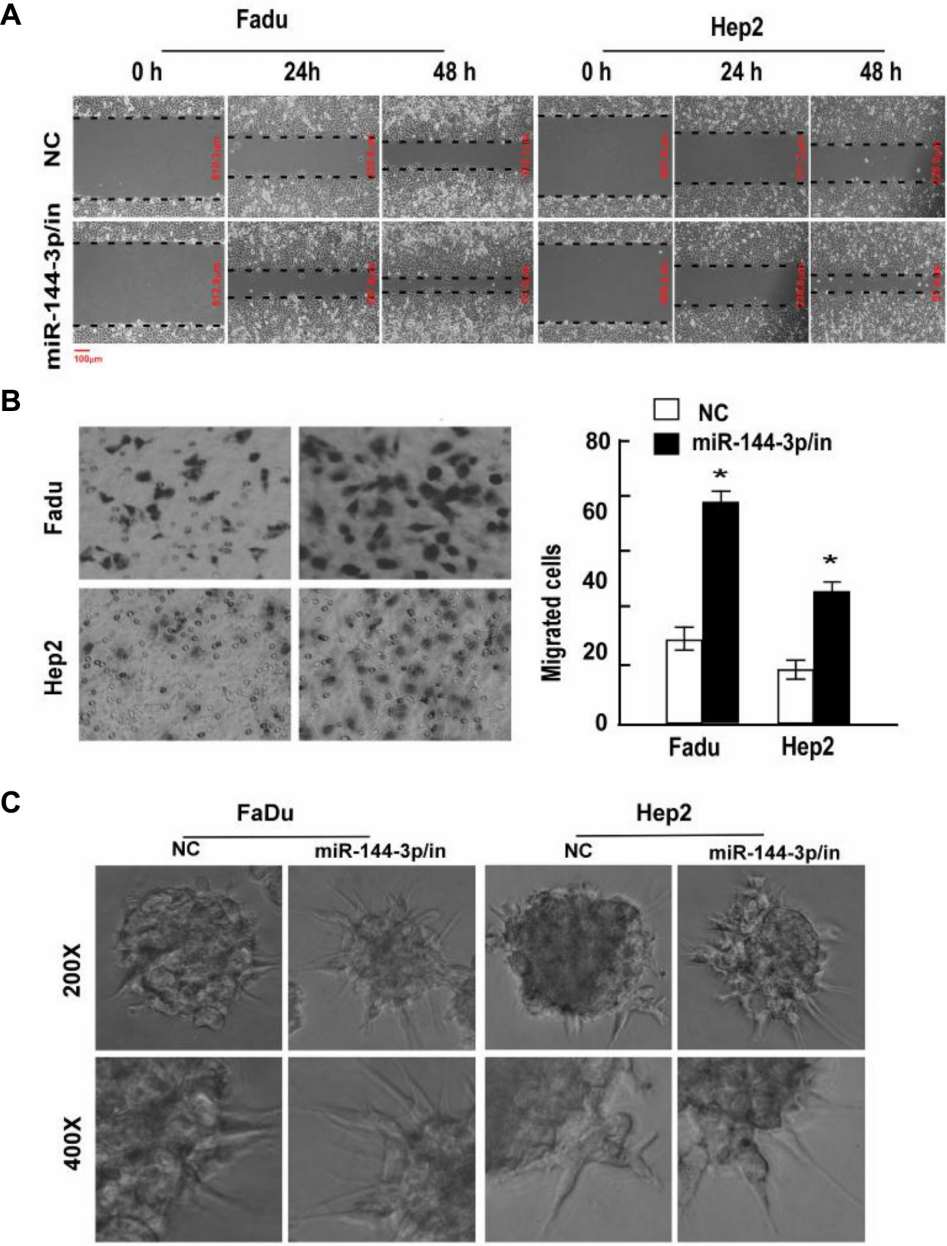
A miR-144-3p inhibitor promotes FaDu and Hep2 cell invasion and migration Following miR-144-3p-inhibitor transfection, FaDu and Hep2 cell invasion and migration was analyzed using a (**A**) wound healing assay, (**B**) transwell invasion assay, and (**C**) 3D-culture test. Values were presented as mean and standard deviation (SD). (B, right panel) *p < 0.05 compared with the NC group.

To confirm the effects of miR-144-3p on cell migration and invasion, FaDu and Hep2 cells were next transfected with a miR-144-3p inhibitor, which significantly reduced miR-144-3p levels (Supplementary Figure 1). As shown in Figure 2A, cells expressing the miR-144-3p inhibitor migrated faster than the NC cells and had greater invasive capacity (*p* ≤ 0.002; Figure 2B). In addition, 3D cultures transfected with a miR-144-3p inhibitor had a greater number of longer cellular processes as compared to the NC group (Figure 2C). Taken together, these results suggest that miR-144-3p inhibits cell migration, invasion and possibly metastasis *in vivo*.

### 2.2 miR-144-3p inhibits cellular epithelial-mesenchymal transition (EMT)

**Figure 3:**
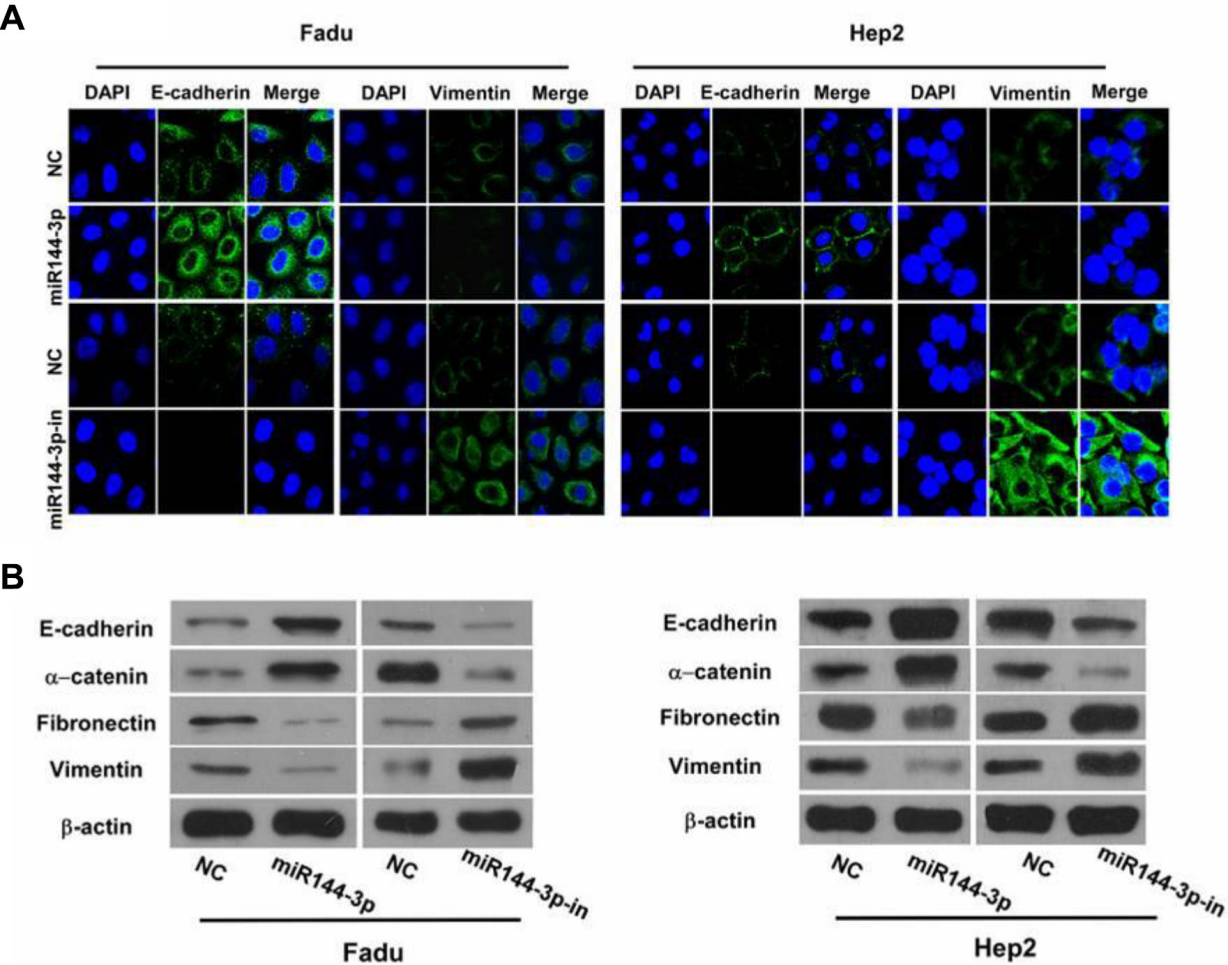
miR-144-3p inhibits cellular epithelial-mesenchymal transition (EMT) (**A**) Immunofluorescence staining of E-cadherin and vimentin (both green) in FaDu cells and Hep2 cells following transfection with miR-144-3p or a miR-144-3p-inhibitor and in NC cells. Cells were counterstained with DAPI (blue) to identify the nuclei. (**B**) Western blot analysis of the epithelial markers, E-Cadherin and α-catenin, and mesenchymal markers, fibronectin and vimentin, in FaDu cells and Hep2 cells following miR-144-3p or miR-144-3p-inhibitor transfection and in NC cells.

Immunofluorescence staining of the epithelial marker, E-cadherin, and the mesenchymal marker, vimentin, in FaDu and Hep2 cells after transfection with miR-144-3p and miR-144-3p inhibitor was next undertaken to examine the role of miR-144-3p on EMT. As shown in Figure 3A, E-cadherin was more abundant in the miR-144-3p-transfected cells and less abundant in cells expressing the miR-144-3p inhibitor. In contrast, vimentin was less abundant in the miR-144-3p-transfected cells and more abundant in cells expressing the miR-144-3p inhibitor.

Western blot analysis of the epithelial markers, E-cadherin and α-catenin, and the mesenchymal markers, fibronectin and vimentin, following miR-144-3p upregulation or inhibition was performed. As shown in Figure 3B, higher expression of the epithelial markers was observed with miR-144-3p upregulation; their expression decreased with miR-144-3p inhibition. Also, the mesenchymal markers were less abundant in the miR-144-3p-transfected cells, but were more abundant with miR-144-3p inhibition (Figure 3B). These findings suggest that miR-144-3p expression prevents EMT in FaDu and Hep2 cells.

### 2.3 miR-144-3p inhibits Hep2 cell growth

**Figure 4:**
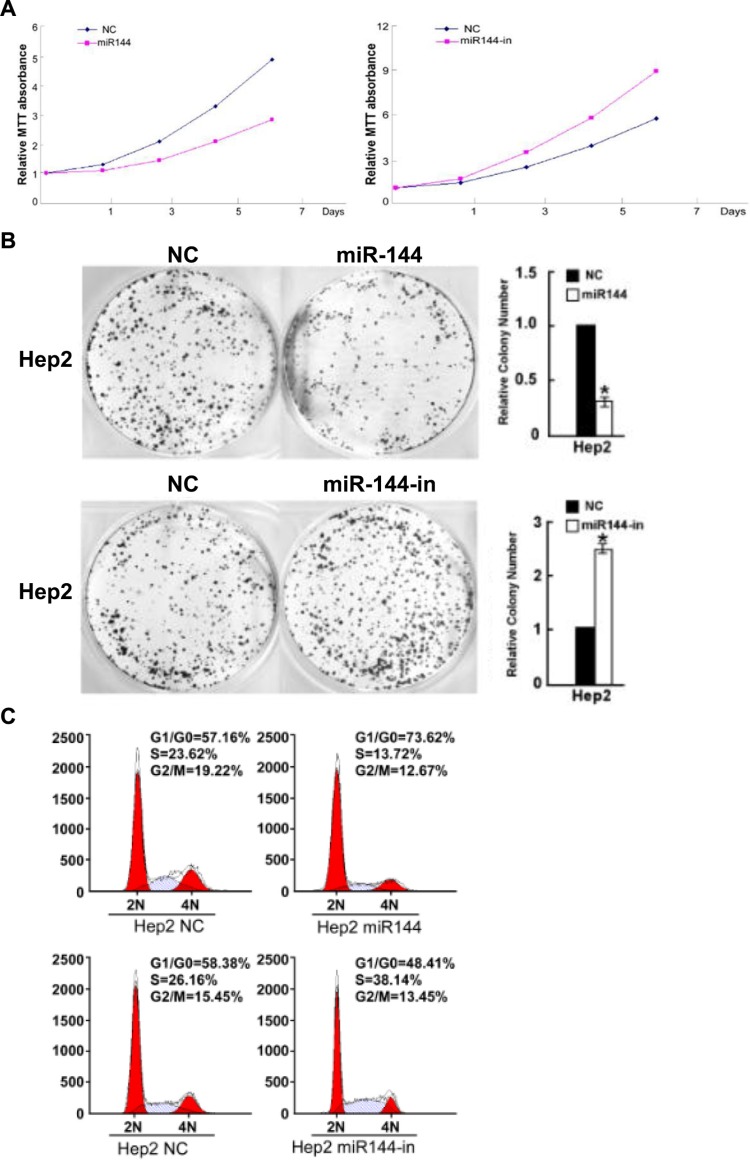
miR-144-3p inhibits Hep2 cell growth Hep2 cell (**A**) proliferation (**B**) colony formation and (**C**) cell cycle progression were decreased with miR-144-3p and increased with its inhibition.

The impact of miR-144-3p on Hep2 cell growth was next assessed using cell proliferation, colony formation and cell cycle progression analyses. As shown in Figure 4A, the proliferation of Hep2 cells transfected with miR-144-mimics was significantly reduced as compared with the control (*F* = 124.055, *p* < 0.001). In contrast, after transfection with a miR-144-inhibitor, the proliferation of Hep2 cells increased significantly (*F* = 702.700, *p* < 0.001). Similarly, transfection with miR-144-3p-mimics significantly reduced the number of colonies formed by Hep2 cells relative to the control group (*t* = 26.361, *p* = 0.000); miR-144-3p-inhibitors increased the number of colonies formed by Hep2 cells (*t* = −24.200, *p* = 0.000; Figure 4B). As shown in Figure 4C, transfection with miR-144-3p mimic significantly increased the proportion of cells in the G0/G1 phase (73.62% vs. 57.16%) and reduced the proportion of cells in the G2/M phase (12.67% vs. 19.22%) as well as the S phase (13.72% vs. 23.62%). After transfection with a miR-144-3p inhibitor, the proportion of cells in the G0/G1 phase increased (48.41% vs. 58.38%); the proportion in the G2/M phase decreased (13.45% vs. 15.45%), and those in the S phase increased (26.16% vs. 38.14%; Figure 4C). Taken together, these data suggest that miR-144-3p inhibits the proliferation of Hep2 cells.

### 2.4 miR-144-3p binds to ETS-1 3′UTR to downregulate ETS-1

**Figure 5:**
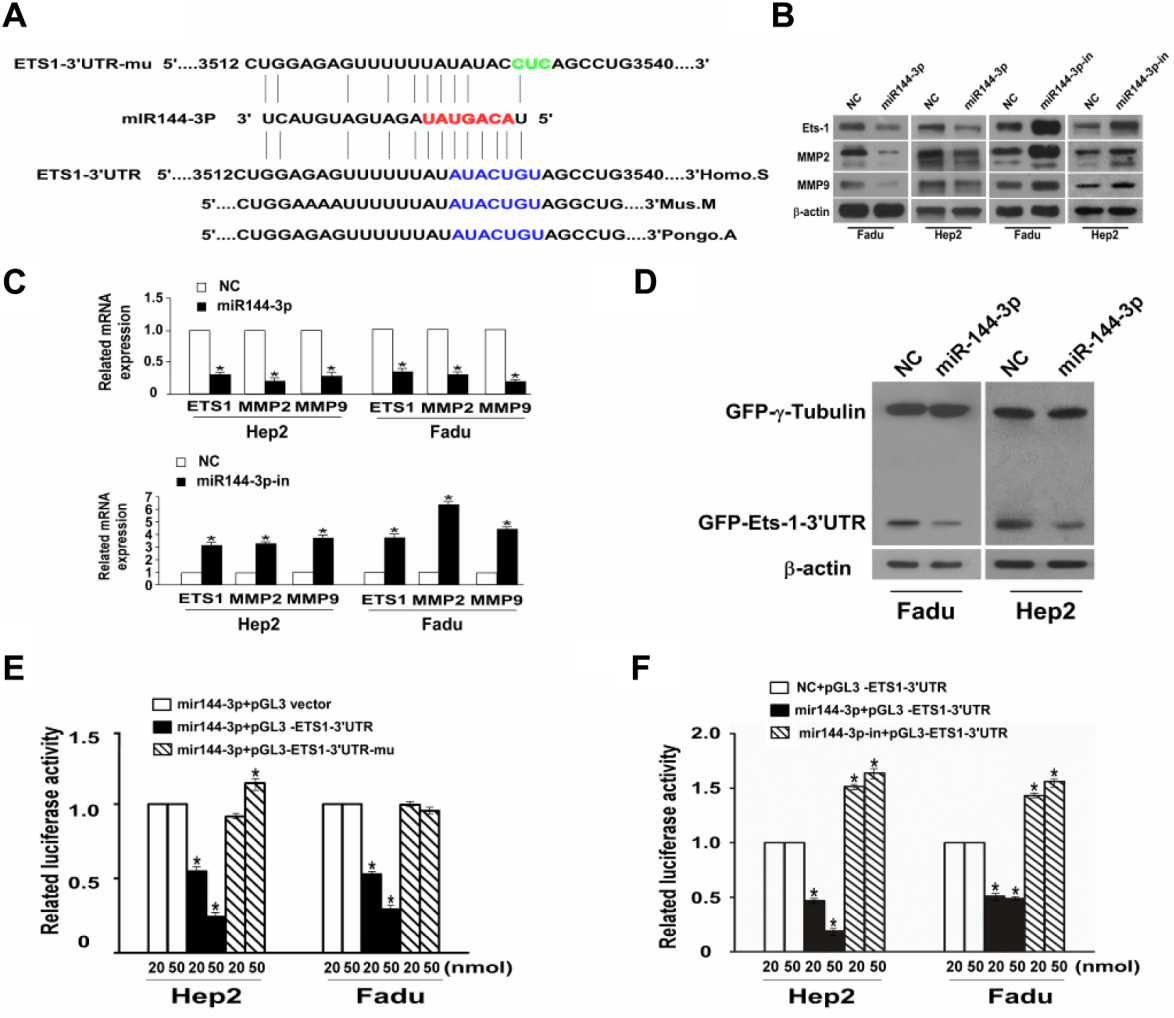
miR-144-3p binds to the 3′UTR of ETS-1 to downregulate its expression together with downregulation of MMP2 and MMP9 (**A**) miR-144-3p binds to the predesigned site of ETS-1 3′UTR. (**B**) Western blot analysis of ETS-1, MMP2, and MMP9 expression in FaDu cells and Hep2 cells following transfection with miR-144-3p or a miR-144-3p-inhibitor and in NC cells. β-actin served as an internal reference. (**C**) qPCR showing relative mRNA expression of ETS-1, MMP-2, and MMP-9. (**D**) Western blot assay for GFP-ETS1-3′UTR after miR-144-3p transfection. (**E**, **F**) Dual luciferase reporter gene analysis of ETS1-3′UTR. Values were presented as mean and standard deviation (SD). (E) *p* < 0.05 compared with the *miR-144-3p + pGL3 vector (20), ^†^miR-144-3p + pGL3 vector (50), ^‡^miR-144-3p + pGL3-ETS1-3′UTR (20), ^§^miR-144-3p + pGL3-ETS1-3′UTR (50), and ||miR-144-3p + pGL3-ETS1-3′UTR-mu (20) groups. (F) *p* < 0.05 compared with *NC + pGL3-ETS1-3′UTR (20), ^†^NC + pGL3-ETS1-3′UTR (50), ^‡^miR-144-3p + pGL3-ETS1-3′UTR (20), ^§^miR-144-3p + pGL3-ETS1-3′UTR (50), and ^||^miR-144-3p + pGL3-ETS1-3′UTR-mu (20).

We previously identified ETS-1 as a putative target of miR-144-3p [10]. Given that ETS-1 can induce the invasion and migration of rat C6 glioma cells [18], we next analyzed whether it was a miR-144-3p target. Analysis of the ETS-1 3′-UTR revealed a putative miR-144-3p target site (Figure 5A). As shown in Figure 5B, Western blot analyses of ETS-1 from FaDu and Hep2 cell lysates showed that *ETS-1* was less abundant in cells overexpressing miR-144-3p. In contrast, ETS-1 was more abundant in cells expressing a miR-144-3p inhibitor as compared to NC cells. Similarly, Western blot analyses of GFP-ETS-1-3′UTR showed that it was less abundant in the miR-144-3p-transfected cells and more abundant with miR-144-3p inhibition as compared to NC cells (Figure 5C). These studies suggest that miR-144-3p downregulates ETS-1 protein expression.

Dual luciferase reporter gene analyses of *ETS-1*-3′UTR in both FaDu and Hep2 cells revealed that the mean luciferase activity was significantly lower in the mir-144-3p + pGL3-ETS-1-3′UTR group compared to the mir-144-3p + pGL3 vector group at both 20 or 50 nmol assay concentrations (all *p* < 0.001; Figure 5D). However, no such downregulation in reporter activity was seen with the mir-144-3p + pGL3-ETS1-3′UTR-mu group (Figure 5D). Similarly, as compared to NC + pGL3-ETS-1-3′UTR group, relative luciferase activities were significantly reduced in the mir-144-3p-in + pGL3-ETS1-3′UTR-mu groups at either the 20 or 50 nmol assay concentrations (all *p* < 0.001; Figure 5E). Luciferase activity was significantly higher in the mir-144-3p-in + pGL3-ETS-1-3′UTR-mu groups compared to the NC + pGL3-ETS-1-3′UTR groups (all *p* < 0.001). These findings confirm that miR-144-3p downregulates ETS-1 protein expression by targeting its 3′-UTR.

The impact of miR-144-3p on the expression of matrix metalloproteinases (MMPs), which are regulators of extracellular matrix composition as well as cell invasion and migration, was next examined. As shown in Figure 5B and 5C, MMP2 and MMP9 protein and mRNA expression were reduced in FaDU or Hep2 cells overexpressing miR-144-3p. In contrast their expression was increased with transfection of a miR144-3p inhibitor.

### 2.5 ETS-1 silencing inhibits cellular invasion and migration *in vitro*, and tumor proliferation *in vivo*

**Figure 6:**
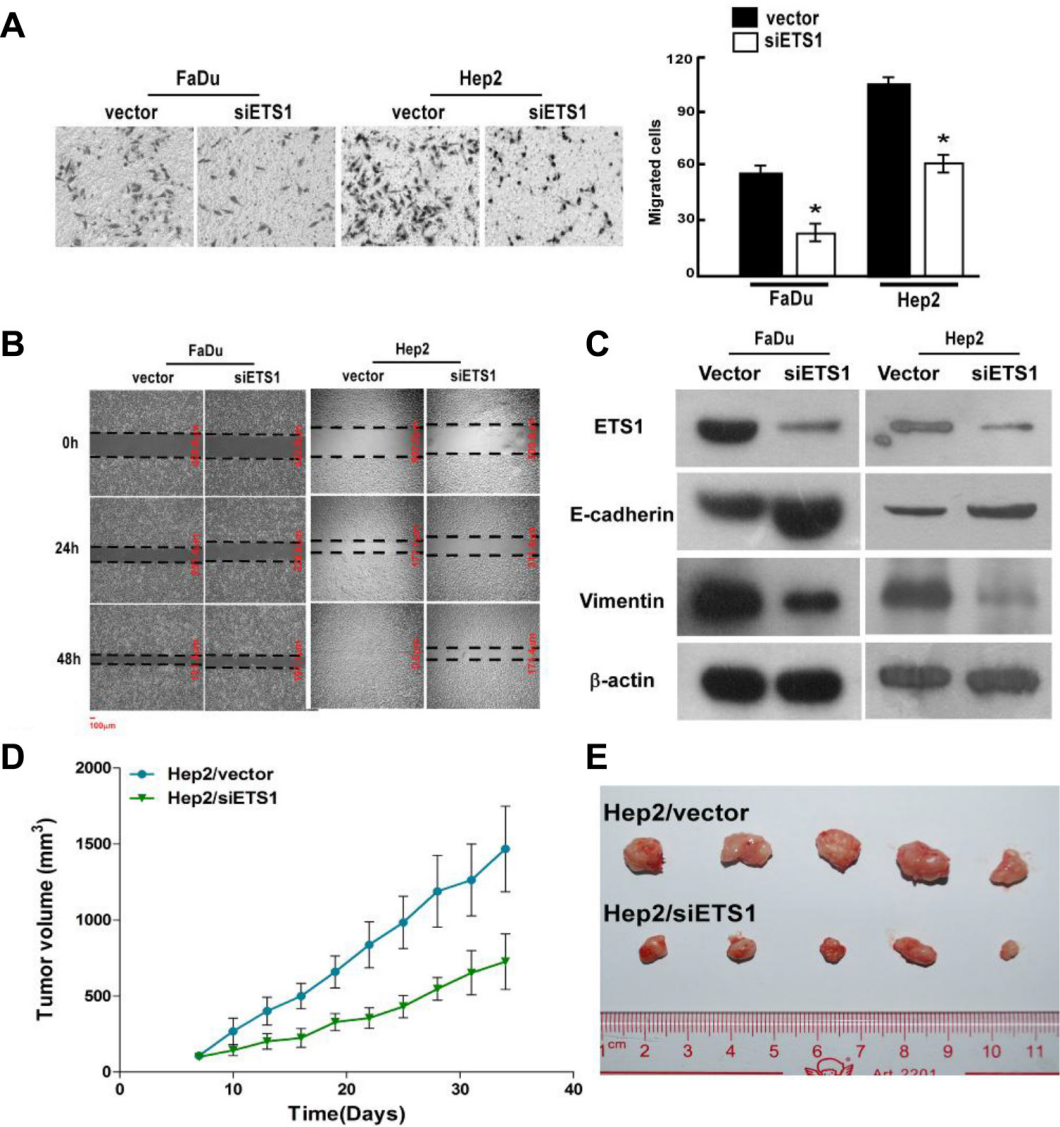
ETS-1 silencing inhibits FaDu and Hep2 cellular invasion and migration *in vitro*, and Hep2 tumor growth *in vivo* After ETS-1 silencing, FaDu and Hep2 cells were analyzed for migration and invasion using (**A**) Transwell invasion and (**B**) scratch wound healing assays. (**C**) Western blot analysis showed that ETS-1 silencing increased the expression of the epithelial marker, E-Cadherin, and reduced the expression of the mesenchymal marker, vimentin, in both FaDu and Hep2 cells. (**D**) Xenograft tumors were established with Hep2/vector and Hep2/siETS1 cells, and tumor volume was measured over time. (A, D) Values were presented as mean and standard deviation (SD). *p < 0.05 compared with (A) Hep2/vector (right panel) and (D) Hep2/siETS1.

To evaluate the effects of ETS-1 downregulation, we next silenced its expression in FaDu and Hep2 cells. As shown in Figure 6A, significantly fewer FaDu/siETS-1 and Hep2/siETS-1 cells migrated as compared to the corresponding vector-transfected control cells (*p* < 0.001), which was confirmed in a wound healing assay using both cell lines (Figure 6B). In addition, suppression of ETS-1 increased the expression of E-cadherin and reduced the expression vimentin (Figure 6C). This suggests that the inhibition of FaDu and Hep2 cellular invasion and migration by ETS-1 silencing occurs via suppression of EMT.

To evaluate the significance of ETS-1 silencing *in vivo*, xenograft tumors of Hep2/vector and Hep2/siETS1 cells were analyzed. As shown in Figure 6D and 6E, Hep2/siETS-1 tumors were significantly smaller from day 10 to the end of the study than those in the Hep2/vector group (all *p* ≤ 0.019). Thus, ETS-1 silencing inhibits tumor growth.

### 2.6 Comparison of ETS-1 expression in normal adjacent and tumor tissue from LSCC patients

**Figure 7:**
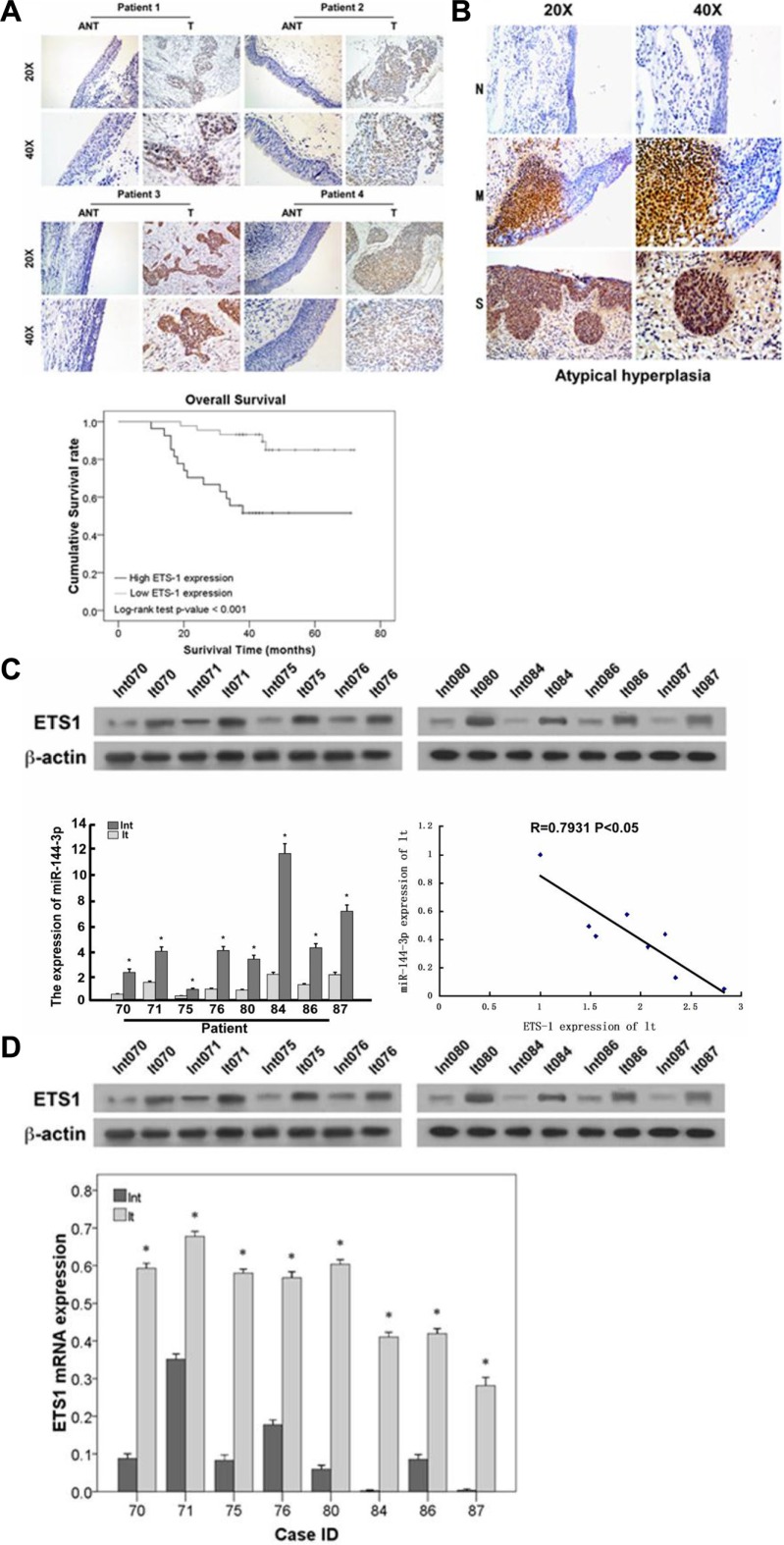
ETS-1 expression increases with LSCC progression and negatively correlates with prognosis (**A**) Immunohistochemistry analysis of ETS-1 in LSCC patients. Patients with relatively lower ETS-1 expression had significantly higher survival rates compared to those with higher ETS-1 expression (*p* < 0.001). (**B**) ETS-1 expression in severe dysplasia laryngeal mucosa (S), mild/moderate laryngeal dysplasia laryngeal mucosa (M), and normal laryngeal mucosa (N). (**C, D**) Comparison of ETS-1 and miR-144-3p levels in LSCC patient samples showing their negative correlation (*p* < 0.05). (C) mRNA and (D) protein expression in adjacent normal and laryngeal carcinoma tissues. Lnt: adjacent normal tissues, lt: laryngeal carcinoma tissues. Values were presented as mean and standard deviation (SD). *p < 0.05 compared with adjacent normal tissue.

We previously showed that miR-144-3p was downregulated in LSCC [10], which may result in increased expression of its targets, including ETS-1. Therefore, we next determined ETS-1 expression in LSCC patient tissues. As shown in Figure 7B, ETS-1 expression increased from normal laryngeal mucosa to mild/moderate dysplasia laryngeal mucosa and severe dysplasia laryngeal mucosa, suggesting that its expression increased with disease progression. Subsequent analysis of eight fresh frozen tissues from LSCC patients undergoing resection revealed that ETS-1 mRNA (Figure 7C) and protein (Figure 7D) expression were significantly higher in the tumor tissues as compared to the adjacent normal counterparts (all *p* ≤ 0.004). Analysis of ETS-1 levels in both the laryngeal carcinoma and adjacent normal tissues revealed that it was negatively correlated with miR-144-3p levels (*p* < 0.05; Figure 7C).

### 2.7 ETS-1 is highly expressed in LSCC and is negatively correlated with prognosis

ETS-1 expression level was next determined in the tumors obtained from patients receiving surgery for LSCC. As shown in Figure 7A, patients with relatively lower ETS-1 expression, as detected by immunohistochemistry, had significantly higher survival rates compared to those with greater ETS-1 expression (*p* < 0.001). These results indicate that ETS-1 could have a direct correlation with the progression of LSCC and could, therefore, be an important biomarker and/or therapeutic target.

## 3 DISCUSSION

miR-144, which targets ETS-1, is significantly downregulated in LSCC [10]. In the present study, a series of experiments were conducted to investigate the role of miRNA-144-3p in LSCC. Our study for the first time found that miRNA-144-3p could suppress ETS-1 expression, reducing the EMT of LSCC cells to inhibit their invasion and metastasis. In addition, ETS-1 was highly expressed in LSCC and negatively associated disease progression and overall patient survival. Thus, miRNA-144-3p and ETS-1 may represent new therapeutic targets for LSCC.

miR-144 plays crucial roles in the occurrence, development, invasion and metastasis of some cancers. A meta-analysis of 28 studies on the microRNA profiling of 4000 cancer and adjacent normal tissues (including 33 controls) confirmed that miR-144 was closely related to liver, lung and prostate cancers [11]. Although it is downregulated in some cancers, including colorectal cancer [19] and follicular thyroid carcinoma [20], miR-144 can promote the growth of cervical cancer cells [21]. Moreover, in nasopharyngeal cancer, miR-144 was upregulated, and plays an important role in malignant progression [13]. Thus, miR-144 may be cancer-specific, exerting anti-tumor or oncogenic effects, depending on the cancer type. In the present study, miR-144 inhibited the invasion and migration of FaDu and Hep2 cells. In addition, miR-144-3p can inhibit cell cycle progression as well as the proliferation of LSCC cells. However, further studies are necessary to elucidate the mechanisms underlying the effects of miR-144.

EMT represents an important mechanism by which cancer cells of epithelial origin metastasize by transforming into cells with a mesenchymal phenotype via specific programs. EMT is characterized by the reduced expression of cell adhesion molecules (e.g., E-cadherin), transformation of cytokeratin (a component of cytoskeleton) into vimentin and altered cellular morphology, which are characteristic of mesenchymal cells. Epithelial cells undergoing EMT lose their connection to the basement membrane, which increases their invasive potential; they also become resistant to apoptosis and can degrade extracellular matrix. Thus, EMT is an important biological process by which cancer cells of epithelial origin gain migratory and invasion capabilities. In this study, altering miR-144-3p influenced the expression of markers of EMT, such as E-cadherin and vimentin. Thus, we show for the first time that low miR-144 expression could promote the invasion and metastasis of LSCC by inducing EMT.

In the present study, miR-144-3p directly suppressed ETS-1 expression. ETS-1 is a signal-dependent transcription factor that is overexpressed in some cancers [22, 23]. ETS-1 plays important roles in cell proliferation, angiogenesis, and apoptosis; it also regulates the invasion, recurrence and metastasis of cancers [18, 24] by altering cell adhesion and promoting matrix proteolysis [25, 26]. Furthermore, ETS-1 increases angiogenesis and tolerance of cancer cells to hypoxia [27, 28]. In contrast, inhibition of ETS-1 can reduce the proliferation and invasion of cancer cells and prolong the survival of *in vivo* models [18, 22]. Moreover, recent studies have found that EST-1 not only plays an important role in anti-chemoresistance [14] and anchorage-independent growth [15], but it is also a potentially important target for inhibiting cancer cell metastasis [16], suggesting that it may be a potential therapeutic target for cancers. However, the exact role of EST-1 in laryngeal-related cancers was unknown. Our *in vitro* and *in vivo* experiments suggest that EST-1 inhibition could inhibit the growth, invasion and metastasis of LSCC cells by regulating EMT. However, the mechanism underlying ETS-1-induced EMT is still unclear. A recent study revealed that silencing ETS-1 inhibited the expression of vascular endothelial growth factor (VEGF) [29]. Given that EMT is often accompanied by abnormal VEGF expression [30, 31], ETS-1-induced EMT might be associated with VEGF expression; however, the exact mechanism requires further in-depth studies.

The current clinicopathological staging system has limitations in predicting the prognosis of LSCC patients. Although several studies have shown that ETS-1 is an important molecular marker for LSCC prognosis [17], no study has investigated the correlation of ETS-1 with the prognosis, occurrence and development of LSCC. Our findings confirmed that ETS-1 expression was upregulated with LSCC progression. In addition, the ETS-1 expression increased gradually from adjacent normal tissues, mild to moderate dysplasia tissues and severe dysplasia tissues, suggesting that it is associated with the occurrence and progression of LSCC. Furthermore, ETS-1 expression was associated with the overall survival of LSCC patients. Thus, high ETS-1 expression may be predictive of poor prognosis of LSCC patients and, therefore, may represent an independent predictor of LSCC.

Studies have shown that miR-144 can also suppress the expression of the tumor suppressor, phosphatase and tensin homolog (PTEN) [13] and mammalian target of rapamycin (mTOR) [19] and inhibit the growth of colon cancer cells in a Notch-1-dependent manner [32]. Moreover, miR-144 also targets zinc finger X-chromosomal protein (ZFX), and downregulation of miR-144 and consequent upregulation of ZFX expression promotes the bone marrow metastasis of gastric cancer cells [33]. Furthermore, the miR-144-ZFX pathway is involved in non-small cell lung cancer development [34]. Our results also indicate that miR-144-3p may possibly have a similar effect on various types of cancers, through regulating different target genes.

The present study is limited in that exact role of ETS-1 in LSCC invasion remains to be determined. Although, the effects of miR-144-3p on reducing cell invasion and migration may be mediated at least in part through ETS-1 expression, the exact mechanism is still unclear and warrants further in-depth investigation. Therefore, further studies will include rescue experiments to definitively show that the impact of miR-144-3p on cell migration and metastasis is mediated through ETS-1. In addition, further experiments will assess the impact of miR-144-3p mimics and its inhibition *in vivo* as well as examine if there is a correlation between LSCC progression or patient prognosis and miR-144-3p expression level.

Taken together, both our *in vivo* and *in vitro* experimental results demonstrated that miR-144-3p may target ETS-1 to inhibit the invasion and metastasis of LSCC via suppressing EMT. Given that ETS-1 is highly expressed in LSCC and negatively associated with LSCC patient survival, it may represent an important prognostic marker and possible therapeutic target.

## 4 MATERIALS AND METHODS

### 4.1 Cell culture

The human head and neck squamous cell carcinoma lines, FaDu, and Hep2 (both from American Type Culture Collection, Manassas, VA, USA), were selected as they are the available laryngeal cell lines in China. Cells were maintained in DMEM medium (Invitrogen, Carlsbad, CA, USA) supplemented with 10% fetal bovine serum (HyClone, Logan, UT, USA) and were maintained in a humidified incubator at 37°C with 5% CO_2_.

### 4.2 Transient transfection

Hep2 cells were inoculated onto 6-well plates (4 × 10^5^ cells/well) in RPMI 1640 medium containing 10% FBS without antibiotics at 37°C. Once the cells reached 70–80% confluence, they were transfected with 100 nM miR-144-3p mimic, miR-144-3p inhibitor, or negative control (NC) (Guangzhou Ruibo Biotech) in 2 mL serum-free total reaction volume with Lipofectamin^®^ 2000 reagent (Invitrogen, Carlsbad, CA, USA). After 6 h, the medium was replaced with medium containing 10% FBS. The cells were lysed 48 h later, and protein and RNA were collected.

### 4.3 Study participants

Tumor samples, including 71 paraffin-embedded tissue biopsies for immunohistochemistry analysis and eight fresh frozen biopsies, were obtained from patients receiving surgery for LSCC in the Division of Otolaryngology, Guangdong General Hospital from January 2007 to November 2008. Patient follow-up was undertaken every three months via phone, mail or outpatient visit. Overall survival was defined as the time between the date of diagnosis and that of patient death or the end of the study period. Informed consent was obtained from all participants. This study was approved by the ethics committee of Guangdong General Hospital.

### 4.4 Immunofluorescence analysis

Immunofluorescence analysis was undertaken as described previously [10]. Briefly, the cells were grown on coverslips, incubated with primary antibodies against fibronectin and vimentin (Cell Signaling Technology, Beverly, MA, USA) and then incubated with rhodamine-conjugated or FITC-conjugated goat antibodies against rabbit or mouse IgG (Jackson Immuno Research Laboratories, West Grove, PA, USA). The coverslips were counterstained with DAPI to identify the nuclei and imaged with a confocal laser-scanning microscope (Axiovert 200 M, Zeiss).

### 4.5 Wound healing assay

Stable Hep2 and FaDu cells were trypsinized and seeded equally into 6-well cell culture plates (5 × 10^5^ cells/well) and maintained for 24 h to reach near confluence. The cells were then serum-starved for 24 h after formation of the cell monolayer. Following starvation, an artificial homogenous wound was created onto the monolayer with a sterile 100 μL pipette tip. After scratching, the cells were washed with serum-free medium. Images of cells migrating into the wound were captured at 0, 12, 24 and 48 h using an inverted microscope (40 ×) (Olymbus).

### 4.6 *In vitro* invasion assay

The invasion assay was performed in a Transwell^®^ chamber (Corning Incorporated, Corning, NY, USA) consisting of 8-μm membrane filter inserts coated with Matrigel^®^. Cells were trypsinized and suspended in serum-free medium after which 1.5 × 10^5^ cells were added to the upper chamber, and the lower chamber was filled with medium containing 10% FBS. After 48 h, the cells on the Matrigel^®^-coated membranes were fixed with 4% paraformaldehyde and stained with hematoxylin. The cells were counted under a microscope using a 100 × objective.

### 4.7 3D morphogenesis assay

After 24-well cell culture plates were coated with Growth Factor Reduced Matrigel^®^ (BD Biosciences, San Jose, CA) and growth medium supplemented with 2% Matrigel, cells were seeded at a density of 10^4^ cells/well. The medium was replaced with 2% Matrigel every 3 to 4 days. Microscopic images were captured at two-day intervals for two to three weeks.

### 4.8 *In vitro* proliferation assay

Hep2 cells, including those transfected with a miR-144 mimic or inhibitor, were cultured at a density of 0.5–1 × 10^4^ cells/mL. After 1, 2, 5, or 7 days, 20 μL of 3-(4,5-dimethylthiazol-2-thiazole)-2,5-diphenyltetrazolium bromide (MTT; 5 mg/mL; Sigma, St. Louis, MO, USA) was added to each well. The plate was wrapped with foil and incubated at 37°C for 4 h. After removing the medium and MTT solution from each well, 150 μL of DMSO was added to each well and incubated for 10 min with a constant shaking to resolve the crystals. The optical density (OD) was measured at 490 nm. Each measurement was performed in triplicate.

### 4.9 Colony formation assay

Hep2 cells, including those transfected with a miR-144 mimic or inhibitor, in logarithmic growth phase were cultured at a density of 1 × 10^3^ cells per 6-well plate. Cells were maintained for 7–10 days after which they were washed three times with 1 × PBS, and 1 mL of methanol fixation solution was added to each well for 10 min with a constant shaking. After staining with hematoxylin, the colonies, defined as an aggregate of > 50 cells, were counted in each well and also photographed. The colony formation rate was calculated as follows: (number of colonies / number of seeded cells) × 100%.

### 4.10 Cell cycle analysis

Cell cycle analysis was undertaken using a Cell Cycle Detection Kit (Beckman Coulter, Brea, CA, USA) following the manufacturer's instructions. Briefly, Hep2 cells, including those transfected with a miR-144 mimic or inhibitor, were digested with EDTA-free trypsin and harvested by centrifugation at 800 rpm for 5 min. After washing twice in PBS, the cells were permeabilized in 70% cold ethanol and incubated at 4°C in the dark overnight. After two washes in PBS, the cell sediment was re-suspended in 500 μL of PBS containing 100 U/mL RNase at 37°C in the dark for 30 min after which 2 mg/mL propidium iodide (PI) was added for an additional 30 min. Cell cycle analysis was assessed by flow cytometry (Beckman) with an excitation wavelength of 488 nm and an emission wavelength of 525 nm.

### 4.11 RNA extraction and real-time quantitative PCR

Total RNA or miRNA from cell lines and primary tumor tissues was extracted using TRIzol^®^ reagent (Invitrogen) according to the manufacturer's instructions. The extracted RNA was dissolved with RNAase-free water that was pretreated with DEPC. cDNA was synthesized from total RNA using PrimeScript RT reagent Kit (TaKaRa, Dalian, China). PCR reactions containing SYBR Premix Ex Taq II (TaKaRa, Dalian, China) were next carried out with the following specific primers: ETS-1 forward, 5′-CTGCAGTGGTGAGGCAAGGA-3′; ETS-1 reverse, 5′-TTCCTGAGTTGCCATCTCATCC-3′; β-actin forward, 5′-TGGCACCCAGCACAATGAA-3′; and β-actin reverse, 5′-CTAAGTCATAGTCCGCCTAGAAGCA-3′. RT-PCR and real-time PCR primers were designed using Primer Express Software V.20 (Life Technologies, Carlsbad, CA, USA). PCR reactions were exposed to the following thermocycling conditions: an initial denaturation step (95°C for 2 min), a cycling step (95°C for 30 s, 60°C for 35 s, repeated for 40 cycles) and melting curve analysis. Expression data were normalized to the geometric mean of the housekeeping gene, *GAPDH*, to control for variability in expression levels. The expression of miRNA was defined based on the threshold cycle (Ct), and relative expression levels were calculated as 2^−[(Ct of miR1440)−Ct of U6]^ after normalization with reference to the expression of U6 small nuclear RNA.

### 4.12 Western blot analysis

Equal amounts of protein were separated by electrophoresis on a 10% SDS polyacrylamide gel and electro-transferred from the gel to a nitrocellulose membrane. After blocking with a 5% milk solution in Tris-buffered saline with Tween (TBS-T) for 1 h, the membrane was incubated with primary antibodies, including anti-ETS1 (BioWorld, Atlanta, GA, USA), anti-E-cadherin, anti-N-cadherin, and anti-vimentin (all from Cell Signaling Technology) as well as anti-fibronectin (Santa Cruz Biotechnology, Santa Cruz, CA, USA) for 1.5 to 2 h at room temperature. Anti-β-actin was used as an internal loading control. After washing with TBS-T, the membrane was incubated with the appropriate secondary antibody for 1 h and examined with enhanced chemiluminescence detection system according to the manufacturer's instructions (Amersham, Arlington Heights, IL, USA).

### 4.13 Luciferase assays

Cells (5 × 10^5^) were seeded in triplicate in 6-well plates and allowed to settle for 24 h. A luciferase plasmid with pGL3-miR-144 mimics (wt/mu) or pGL3-miR-144 inhibitor (wt/mu) was transfected into Hep2 and FaDu cells using the Lipofectamine^®^ 2000 reagent (Life Technologies) according to the manufacturer's recommendation. *Renilla* and firefly luciferase signals were measured 48 h after transfection using the Dual-Luciferase^®^ Reporter Assay Kit (Promega) according to a protocol provided by the manufacturer. Three independent experiments were performed, and the data are presented as the mean ± SD.

### 4.14 Xenograft tumor model

BALB/c-nu mice (four to five weeks of age and weighing 15 to 18 g) were purchased from the Guangdong Experimental Animal Center, and their care was in accord with institutional guidelines. The mice were randomly divided into two groups (*n* = 5/group). One group of mice was inoculated subcutaneously with 5 × 10^6^ Hep2 cells expressing the vector control (Hep2/Vector cells) in the left dorsal flank and with 5 × 10^6^ Hep2 cells expressing ETS-1 (Hep2/ETS-1 cells) in the right dorsal flank. Tumor volume was calculated using the following equation: (L × W2)/2. On day 42, the mice were euthanized, and the tumors were excised, weighed and paraffin-embedded.

### 4.15 Immunohistochemistry analysis

Immunohistochemistry was carried out using ETS1 primary antibodies (dilution 1:600; BioWorld) and DAKO Real EnVision anti mouse/rabbit kit (Dako, Agilent Technologies) following the manufacturer's protocols. Two independent pathologists, blinded to the clinical parameters, conducted the immunoreactivity score (IRS) for ETS-1 expression. The staining results were scored based on the following criteria: (i) percentage of positive tumor cells in the tumor tissue, including 0 (0%), 1 (1–10%), 2 (11–50%) and 3 (51–100%); and (ii) staining intensity, including 0 (no staining), 1 (weak staining = light yellow), 2 (moderate staining = yellow brown), and 3 (strong staining = brown). IRS was calculated as staining intensity score × proportion of positive tumor cells. Cut-off values for ETS-1 were chosen on the basis of a measure of heterogeneity with the log-rank test statistical analysis with respect to overall survival. An optimal cut-off value was identified: tumors with IRS for ETS-1 of ≥ 4 were considered to have high ETS-1 expression and those with ≤ 3 were defined as having low ETS-1 expression.

### 4.16 Statistical analyses

Continuous variables were presented as means and standard deviations (SDs). Independent *t*-tests were used to compare the differences between two groups. One-way ANOVA with Bonferroni post-hoc tests were performed to compare the differences between three or more groups. Data from MTT assay were compared between two groups with repeated measures analysis of variance. Paired-*t* tests were performed to compare the differences between cancer adjacent normal tissues and laryngeal carcinoma tissue as well as the differences in colony formation and cell cycle progression. Kaplan-Meier curve with a log-rank test was used to evaluate the survival rate between patients with high- and low-ETS-1 expressing tumors. SPSS software version 17 (SPSS Inc, Chicago, IL, USA) was used for all statistical analyses. A two sided *p*-value < 0.05 was considered significant.

## SUPPLEMENTARY MATERIALS FIGURE


