# Supplementary material for: miR-144-3p, a tumor suppressive microRNA targeting ETS-1 in laryngeal squamous cell carcinoma
Source: Oncotarget. 2016 Jan 27;7(10):11637–50. doi: 10.18632/oncotarget.7025 (PMC4905499; doi:10.18632/oncotarget.7025)
Supplement: Supplementary file 1 [file oncotarget-07-11637-s001.pdf]

miR-144-3p, a tumor suppressive microRNA targeting ETS-1 in laryngeal squamous cell carcinoma

Supplementary Materials

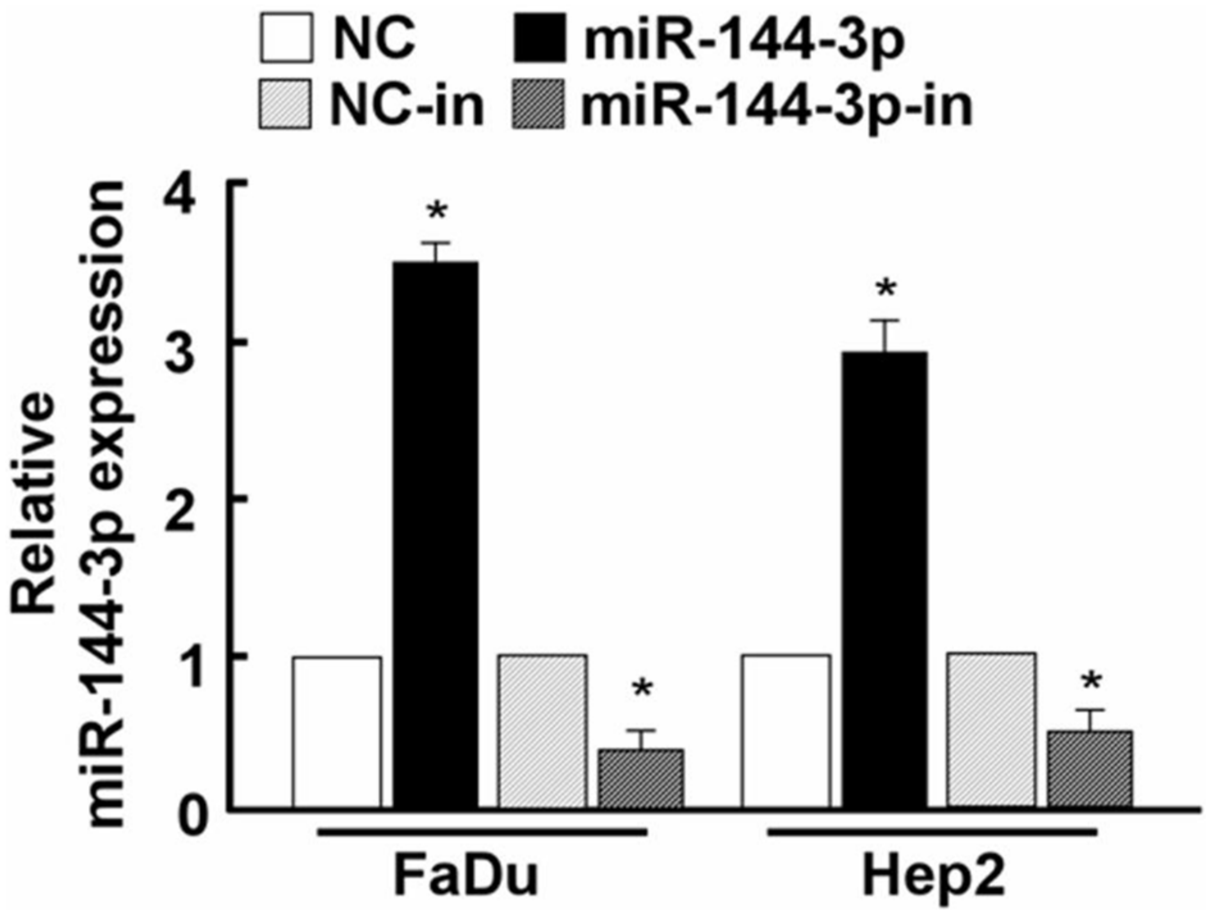

Supplementary Figure S1: Expression of miR-144-3p in FaDu and Hep2 cells following transfection with a miR-144-3p mimic or inhibitor. Relative miR-144-3p expression was determined by qRT-PCR. *$p < 0.05$ compared with NC cells.
